# Imatinib adherence associated clinical outcomes of chronic myeloid leukaemia treatment in Taiwan

**DOI:** 10.1007/s11096-013-9876-7

**Published:** 2013-11-19

**Authors:** Teng-Chou Chen, Li-Chia Chen, Yaw-Bin Huang, Chao-Sung Chang

**Affiliations:** 1 Graduate Institute of Clinical Pharmacy, Kaohsiung Medical University, Kaohsiung, Taiwan; 2 Division for Social Research in Medicines and Health, School of Pharmacy, University of Nottingham, East Drive, University Park, Nottingham, NG7 2RD UK; 3 Division of Hematology-Oncology, Department of Internal Medicine, Kaohsiung Medical University Hospital, Kaohsiung Medical University, Kaohsiung, Taiwan

**Keywords:** Chronic myeloid leukaemia, Imatinib, Persistence - Taiwan, Medication possession ratio

## Abstract

*Background* Since the launch of imatinib, chronic myeloid leukaemia has become a chronic condition requiring costly long-term treatment. Emerging evidence from several short-term studies has raised concerns on the detrimental clinical outcomes and waste of resources associated with poor adherence to imatinib. *Objective* This study aims to evaluate the effects of long-term imatinib adherence on clinical treatment responses and mortality. *Setting* This retrospective cohort study was conducted in a medical centre in southern Taiwan. *Method* Chronic myeloid leukaemia patients who were prescribed for more than 1 month of imatinib were identified and their medical charts were reviewed from the first date of imatinib prescription to the last date of medical record or upon patients’ death. Patients’ basic characteristics, imatinib prescriptions, results of laboratory tests, episodes of imatinib-related side effects and mortality rate were recorded. *Main outcome measure* Participants’ basic characteristics, medication possession ratio and their mortality rate; the association between the medication possession ratio and treatment responses. *Results* Of the 119 included patients, the mean follow-up time was 3.9 ± 2.9 patient-years and the mean medication possession ratio was 89.7 %. At the 18th month of imatinib treatment, 67.2, 54.3 and 34.5 % patients achieved complete cytogenetic, major molecular and complete molecular responses, respectively. There was a significant difference in the 4-year survival rate between the adherence (n = 87) and non-adherence (n = 32) groups (91 vs. 72 %; *p* = 0.0076). Logistic regression analysis revealed that imatinib adherence was the only factor that significantly influenced the 18th month complete cytogenetic response [odds ratio (OR) 11.6; 95 % confidence interval (CI) 1.7, 114.7; *p* = 0.0131] and major molecular response (OR 5.1; 95 % CI 1.1, 26.8; *p* = 0.0351). Cox regression analysis demonstrated that a medication possession ratio greater than 90 % significantly reduced the mortality risk (hazard ratio 0.1; 95 % CI 0.01, 0.60; *p* = 0.0118). *Conclusion* Chronic myeloid leukaemia patients’ long-term adherence to imatinib is significantly associated with the 18th month treatment responses including the cytogenetic response, molecular response and the long-term survival rate in clinical practice.

## Impact of findings on practice statements

- Imatinib users in Taiwan achieving early-stage treatment targets were more likely to have better long-term outcomes.
- Multiple therapy switches in Taiwanese imatinib users seem to lead to poor adherence and outcomes.

## Introduction

Chronic myeloid leukaemia (CML) is a bone marrow stem cell disorder caused by mutated chromosome (Philadelphia chromosome) and is characterised by the increased growth of premature white cells [1]. The annual incidence rate of CML was approximately 1–2 per 100,000 people, and accounted for 15–20 % of all adult leukaemia patients in Western countries. It occurs in all age groups but is more prevalent with the middle-aged and the elderly, and is slightly more common in males than females [2]. Most CML patients are diagnosed at the chronic phase with relatively mild symptoms, but as the disease progresses to the accelerated and blast phases, hyperleukocytosis, abnormality platelet level, and other crisis systematic symptoms often lead to mortality [3].

Traditional treatments for CML include chemotherapy (such as cytarabine, hydroxyurea), interferon, and hematopoietic stem cell transplantation (HSCT). Although the latter is still the only curative option, the use of this is limited due to the lack of human leukocyte antigen matched donor and the potential chronic graft-versus-host disease [4]. After the launch of tyrosine kinase inhibitors (TKIs, such as imatinib), interferon and chemotherapy are used less frequently due to the limited efficacy and the intolerable adverse effects [4], and hydroxyurea is only used to control leukocytosis.

Since the launch of imatinib, the first TKI soon becomes the first-line of treatment for CML due to the advantages of low toxicity, the route of oral administration and the significant improvement in the survival rate (95.2 %) as demonstrated in clinical trials [5]. In the last decade, this innovative pharmacotherapy has turned CML from a progressive disease with a high mortality rate into a chronic condition. The long-term use of expensive imatinib has also resulted in the increasing cost of CML treatment, and CML has now become one of the most costly diseases [6].

In relation to the increasing therapeutic cost, ensuring medicine adherence and optimal disease control have become challenging issues in the long-term utilisation of imatinib. Guidelines from the National Comprehensive Cancer Network (NCCN) suggest that monitoring indictors for long-term TKI efficacy at the 3rd, 6th, 12th and 18th month of this treatment (Table 1) [7]. However, since no indicator has been established after the 18th month of treatment, a patients’ survival is regarded the only outcome measure [8].

**Table 1:** Definitions of tyrosine kinase inhibitor treatment responses

Treatment response	Month of treatment	Definition
Complete hematologic response (ChR)	3rd	White blood count less than 10 × 10^9^/L, platelet count less than 450 × 10^9^/L and no immature cell
Partial cytogenetic remissions (PCyR)	6th	A reduction of Ph-positive cells to 1–34 %
Complete cytogenetic response (CCyR)	12th	The disappearance of Ph-positive cells (0 % cells with Ph-positive)
Complete cytogenetic response (CCyR)*	18th	The disappearance of Ph-positive cells (0 % cells with Ph-positive)
Complete molecular response (CMR)	18th	Undetectable BCR-ABL transcripts (a fusion gene indicating the mutation point of Ph)
Major molecular response (MMR)	18th	A 3-log reduction in transcript level

These definitions are adopted from the National Comprehensive Cancer Network (NCCN) guideline [7]. The standard treatment target at 18th month can achieve CCyR, but CMR and MMR are regarded as better outcomes

Emerging evidence has raised concern about the detrimental effects associated with poor adherence to oral anticancer drugs, including imatinib [9]. This could worsen treatment outcomes, resulting in treatment failure and substantial waste of healthcare resources [10]. However, previous studies have only evaluated adherence to imatinib on chronic-phase and treatment-naïve CML patients who were predominately recruited from Western countries [11] and followed for less than 1 year. [10–13]. Therefore, evidence for the association between long-term adherence to imatinib and CML survival in Asian populations is still limited.

In Taiwan, the incidence of CML was 1.2 case per 100,000 population from 1998 to 2007, and the mean age of diagnosis was 55.7 years [14]. CML treatment is delivered under the coverage of the Taiwan National Health Insurance (NHI), and imatinib and other second-generation TKIs (including dasatinib and nilotinib) were available for CML patients from 2003 to 2008 respectively. According to the NHI reimbursement policy, imatinib is the first-line of treatment for all patients including those diagnosed at chronic, accelerated or blast phase, and dasatinib and nilotinib are only reserved for patients who are resistant or intolerant to imatinib. Studies on imatinib utilisation and the impacts of adherence to TKIs on CML control in Taiwan are still very limited.

## Aim of the study

This study aimed to measure and evaluate the association between long-term imatinib adherence and the treatment outcomes in a Chinese population.

## Methods

### Study design and cohort

This retrospective cohort study was conducted from May 2011 to March 2012 in a medical centre in southern Taiwan after the ethics approval from the Institutional Review Board of the research centre (reference: IRB-20110160) was granted. This hospital, together with two other medical centres, offer tertiary care for approximately 3.3 million inhabitants in southern Taiwan, and there are around 6,000 outpatients visiting the research centre daily. At the time of research, it was estimated that around 120 CML patients have visited the research centre for treatment.

Hospital electronic administration records and patients’ medical records were used as the research data source. Patients who were diagnosed as Philadelphia chromosome positive (Ph-positive) CML and prescribed imatinib for more than 1 month were identified from the hospitals’ electronic administration records from January 2000 to October 2011. Patients’ medical charts were reviewed by a researcher from the first imatinib prescription date (the index date) to either the data collection date or the date of the last medical record. The duration from the index date to the end of follow-up is defined as the ‘follow-up period’.

### Data collection

A data collection form for recording patients’ demographics, disease characteristics, imatinib utilisation and clinical outcome indicators was designed according to existing literature and oncology expert opinions, and it was piloted on three patients’ charts to ensure its feasibility. The piloting results are also included in the analysis.

Patients’ demographics (age and gender), Charlson comorbidity index [15, 16], records of CML treatments prior to imatinib (interferon, HSCT or second-generation TKIs) and the disease stage when imatinib was initiated were collected. Patients’ imatinib prescription details were followed from the index date to the last prescription record. Other treatments such as HSCT or second-generation TKIs given during the follow-up period were also recorded.

All laboratory test results of biological markers for hematologic, cytogenetic and molecular responses were recorded as the clinical indicators for disease prognosis. Laboratory tests associated with imatinib-related adverse effects, including white blood cell count, platelet, glutamate oxaloacetate transaminase, glutamic pyruvic transaminase, and bilirubin levels were also recorded. Imatinib-related adverse effects, i.e. leukocytopenia, thrombocytopenia and hepatotoxicity were defined following the NCCN guideline [7], and the severity was graded according to the Common Toxicity Criteria developed by the National Cancer Institute of the National Institutes of Health in the US.

### Adherence and outcome measures

The primary outcome measures include imatinib-related adherence, clinical outcomes and mortality. The medication possession ratio (MPR) was obtained by dividing each patient’s ‘total number of days of supply’ of imatinib prescriptions by the ‘prescription duration’ as a proxy for adherence, and a conventional cut-off of less than 90 % was used as a synonym for non-adherence [11]. For patients who never received HSCT or second-generation TKIs prior to imatinib, their mortality rate and the four treatment response criteria (Table 1) recommended by the NCCN [7] were used to measure the clinical outcomes.

### Data analysis

Participants’ basic characteristics, MPR, treatment response and imatinib-related side effects are presented in descriptive statistics. The imatinib treatment pathway of the study cohort is presented in proportion according to different therapies, imatinib utilisation patterns (switched), and follow-up endpoints. Kaplan–Meier analysis and Log-rank tests were used to compare mortality rate between adherence and non-adherence groups for patients who never received HSCT or other second-generation TKIs prior to imatinib.

For patients who had biological markers related to complete cytogenetic response (CCyR), major molecular response (MMR) and complete molecular response (CMR) recorded at the 18th month, covariates associated with achieving treatment responses at the 18th month were evaluated using a logistic regression model. Binary covariates which were evaluated in the model included: whether patients younger than 50 years were male, whether patients’ CCI was equalled to 0, whether patients were at chronic phase when imatinib started, whether patients had an MPR > 90 % and whether patients had imatinib related-side effects. The regression results were presented in odds ratio (OR) and 95 % confidence interval (95 % CI). In addition, Cox regression was used to test the association between covariates and the mortality rate in follow-up period, and the results were presented in hazard ratio (HR) and 95 % CI.

Furthermore, various cut-off points of MPR were used to test the impacts of non-adherence definitions [17]. The significance level was set at *p* < 0.05. All analyses were conducted using JMP version 8.0 (SAS Institute Inc., Cary, NC, USA, 2008).

## Results

### Patient characteristics

Overall, 119 patients with a total follow-up time of 469.2 patient-years (mean 3.9 ± 2.9 years) were included in this study. The majority of the patients were in their middle age (mean 45.7 ± 16.9 years), males (n = 70, 58.8 %), treated with imatinib from the chronic phase of CML (n = 92, 77.3 %) without major co-morbidity (Table 2).

**Table 2:** Characteristics of patients

Characteristic	Total	Adherence^(d)^		*p* value
		Adherence	Non-adherence	
Number of patients (%)	119	87 (73.1 %)	32 (26.9 %)	
Gender				
Male	70 (58.8 %)	53 (60.9 %)	17 (53.1 %)	0.4437
Age				
Mean ± SD^(a)^	45.7 ± 16.9	45.3 ± 16.9	46.7 ± 17.4	0.6923
Median (Q1, Q3)	46 (31.5, 58.8)	44 (31, 58.3)	48.5 (33.3, 60.8)	
Over 50 years	47 (40.5 %)	36 (41.9 %)	11 (36.7 %)	0.6178
Follow-up time (year)				
Total (patient-years)	469.2	364.3	104.9	
Mean ± SD^(a)^	3.9 ± 2.9	4.2 ± 2.9	3.3 ± 2.8	0.1463
Median (Q1, Q3)	3 (1.5, 6)	3.8 (1.6, 6.3)	2.1 (1.5, 5.2)	
CCI score (%)^(b)^				0.6800
0	78 (65.5 %)	57 (65.5 %)	21 (65.6 %)	
1	29 (24.4 %)	20 (23 %)	9 (28.2 %)	
2	9 (7.6 %)	8 (9.2 %)	1 (3.1 %)	
3	3 (2.5 %)	2 (2.3 %)	1 (3.1 %)	
Disease stages at imatinib initiating				0.6728
Chronic phase	92 (77.3 %)	68 (78.2 %)	24 (75.0 %)	
Accelerated phase	21 (17.6 %)	14 (16.1 %)	7 (21.9 %)	
Blast phase	6 (5.1 %)	5 (5.7 %)	1 (3.1 %)	
Prior treatments				
Interferon	37 (31.1 %)	21 (24.1 %)	16 (50.0 %)	0.0069*
HSCT^(c)^	2 (1.7 %)	2 (2.3 %)	0	0.3843
Second-generation TKIs	1 (0.8 %)	1 (1.1 %)	0	0.5425
Hydroxyurea	79 (66.4 %)	55 (63.2 %)	24 (75 %)	0.2277
Prior treatment-naïve patients	30 (25.2 %)	24 (28.6 %)	6 (18.8 %)	0.3147
Time to initiate imatinib initiating (year)				
Mean ± SD^(a)^	0.7 ± 1.7	0.6 ± 1.4	1.3 ± 2.5	0.0845
Median (Q1, Q3)	0.1 (0.01, 0.4)	0.1 (0.01, 0.2)	0.1 (0.01, 2.1)	
Imatinib-related side effects				
Grade II leukocytopenia	22 (18.5 %)	10 (11.5 %)	12 (37.5 %)	0.0012*
Grade II thrombocytopenia	49 (41.2 %)	29 (33.3 %)	20 (62.5 %)	0.0042*
Grade II hepatotoxicity	11 (9.2 %)	7 (8 %)	4 (12.5 %)	0.7540

^(a)^ *SD* standard deviation. ^(b)^ *CCI* Charlson comorbidity index. ^(c)^ *HSCT* hematopoietic stem cell transplantation. ^(d)^Adherence: patients whose imatinib medication possession ratio (MPR) ≥ 90 %; non-adherence: patients whose imatinib medication possession ratio (MPR) < 90 %

Prior to imatinib treatment, 39 (32.8 %) patients had received other treatments for CML, and the majority received interferon (n = 37, 31.1 %); only a few patients received HSCT (n = 2, 1.7 %) and second-generation TKIs (n = 1, 0.8 %). In addition, 79 (66.4 %) patients had received hydroxyurea for controlling leukocytosis. Therefore, there were only 30 (25.2 %) treatment naïve patients. During the follow-up period, 22 (18.5 %), 49 (41.2 %) and 11 (9.2 %) patients experienced Grade II leukocytopenia, Grade II thrombocytopenia and hepatotoxicity respectively (Table 2).

There was no significant difference in patients’ characteristics between the adherence and non-adherence groups, but a higher proportion of non-adherence patients who were found to have received interferon (24.1 vs. 50 %, *p* = 0.0069) prior to imatinib treatment and experienced grade II leukocytopenia (11.5 vs. 37.5 %, *p* = 0.0012) and thrombocytopenia (33.3 vs. 62.5 %, *p* = 0.0042) (Table 2).

### Imatinib utilisation pattern

**Fig. 1:**
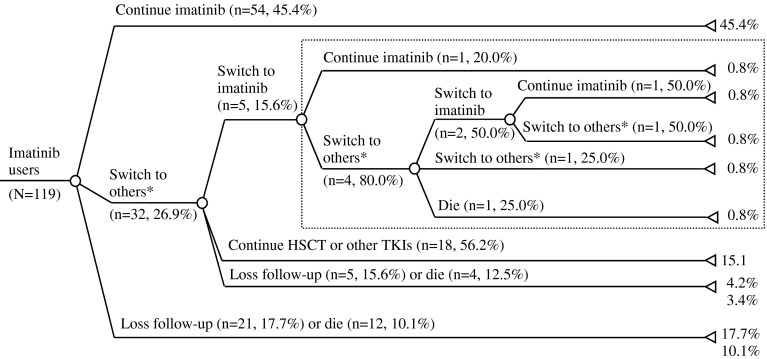
Chronic myeloid leukaemia patients’ utilisation pattern of imatinib and other treatments. *Other treatments include either hematopoietic stem cell transplantation (HSCT) or other tyrosin kinase inhibitors (TKIs). The *dot rectangle* highlights the multiple switches of imatinib treatment

Of the 119 patients, 17 (14.3 %) patients died and 26 (21.8 %) patients missed the follow-up appointments (stopped visiting the research site), whereas 56 patients (47.1 %) were receiving imatinib and 20 (16.8 %) patients discontinued imatinib and switched to second-generation TKIs or HSCT at the end of the follow-up. Overall, 87 (73.1 %) patients only used imatinib and 32 (26.9 %) patients had switched to other treatments (HSCT or other TKIs) during the study period. For the 32 patients who had switched from imatinib to other treatments, a small number of patients (n = 5, 15.6 %) switched back to imatinib treatment, but only 2 (40.0 %) of those active patients kept using imatinib by the end of the follow-up (Fig. 1).

### Adherence to imatinib

**Fig. 2:**
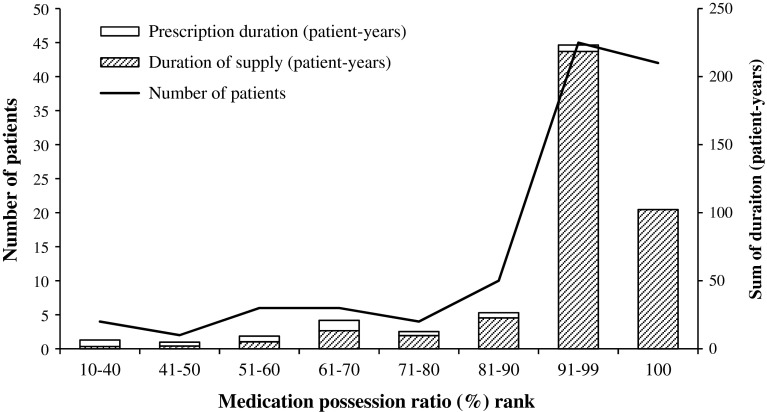
Number of patients and patient-years in each medication possession ratio rank

The median imatinib prescription duration was 2.1 (range 0.2–11.1) years. Most patients (n = 57, 47.9 %) used imatinib for less than 2 years, while 35 (29.4 %) and 10 (8.4 %) patients used imatinib for over 5 and 9 years, respectively. Patients were generally adherent to imatinib, the median MPR of the 119 patients was 98.3 % (range 12.6–100 %), and it was more than 90 % for 87 (73.1 %) patients and 100 % for 41 (34.5 %) patients. However, there was still a small proportion of patients (n = 12, 10.1 %) whose MPR was lower than 60 % (Fig. 2).

### Treatment responses and survival rate

**Fig. 3:**
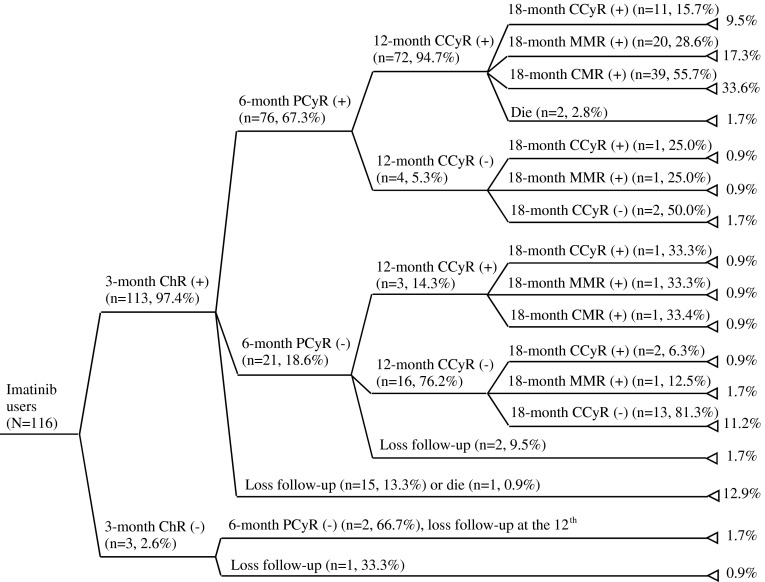
Proportion of imatinib users achieving treatment responses at various points up to the 18th month of treatment. *ChR* complete hematologic response, *PCyR* partial cytogenetic response, *CCyR* complete cytogenetic response, *MMR* major molecular response, *CMR* complete molecular response

**Fig. 4:**
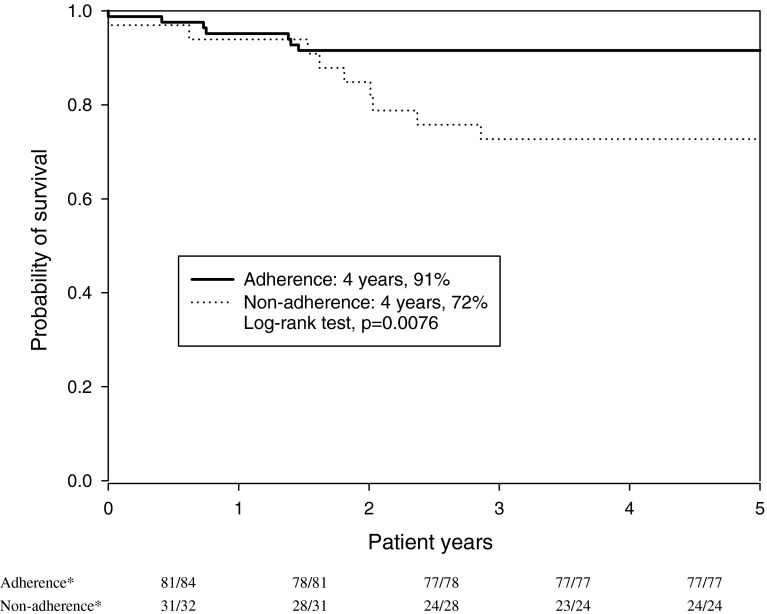
Kaplan–Meier survival curves comparing the probability of survival between adherence and non-adherence groups. Adherence: patients’ imatinib medication possession ratio (MPR) > 90 %; non-adherence: patients’ MPR ≤ 90 %. * Numerator: number of patients survived in the patient-year; Denominator: number of patients contributing to the patient-year

The short-term response to imatinib treatment for the 116 patients who never received HSCT or second-generation TKIs prior to imatinib were generally satisfactory, 113 (97.4 %), 76 (65.5 %) and 75 (64.7 %) patients achieved ChR, PCyR and CCyR at the 3rd, 6th and 12th month of imatinib treatment, respectively. At the 18th month of imatinib treatment, 78 (67.2 %), 63 (54.3 %) and 40 (34.5 %) patients achieved CCyR, MMR and CMR (Fig. 3). Sixteen of the 116 patients died during the follow-up period and the overall 4-year survival rate was 86.2 %. There was a significant difference in terms of the 4-year survival rate between the adherence (76/83; 91 %) and non-adherence group (24/33; 72 %) (*p* = 0.0076) (Fig. 4).

### Adherence associated treatment responses and mortality

The median MPR for the 87 patients who had results of biological markers for CCyR, MMR and CMR recorded within 18 months of imatinib treatment was 99.4 % (range 10.4–100 %). Logistic regression analysis revealed that imatinib adherence (i.e. MPR > 90 %) was the only factor that might have significantly influenced the 18th month CCyR and MMR rates, despite the wide range of ORs (11.6; 95 % CI 1.7, 114.7; *p* = 0.0131 and 5.1; 95 % CI 1.1, 26.8; *p* = 0.0351). None of the covariates found associated with the CMR rate at the 18th month of imatinib treatment. Furthermore, results of Cox regression demonstrated that an MPR greater than 90 % could significantly reduce the mortality risk (HR 0.1; 95 % CI 0.01, 0.6; *p* = 0.0118). On the contrary, it was found that experience of grade II thrombocytopenia was associated with increased mortality (HR: 8.1, 95 % CI 1.4, 65.9; *p* = 0.0223).

**Fig. 5:**
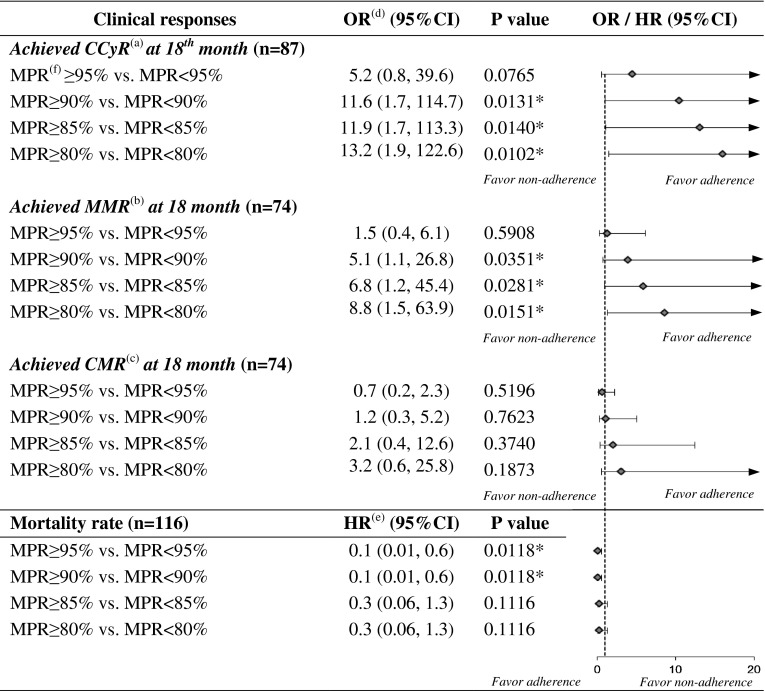
Sensitivity analysis for the influences of various medication possession ratio cut-offs on clinical outcomes and mortality. ^(a)^ *CCyR* complete cytogenetic response. ^(b)^ *MMR* major molecular response. ^(c)^ *CMR* complete molecular response. ^(d)^ *OR* odds ratio. ^(e)^ *HR* hazard ratio. ^(f)^ *MPR* medication possession ratio. *95* *% CI* 95 % confidence interval

The sensitivity analysis assessing various cut-off points of MPR to define adherence indicated that adherence to imatinib was associated with a higher proportion of patients achieving CCyR at the 18th month when adherence was defined as MPR over 90, 85 and 80 %, with OR at 11.6 (95 % CI 1.7, 114.7; *p* = 0.0131), 11.9 (95 % CI 1.7, 113.1; *p* = 0.0140) and 13.2 (95 % CI 1.9, 122.6; *p* = 0.0102) respectively. Similarly, those MPR cut-offs were associated with a higher proportion of patients achieving MMR at the 18th month, with OR at 5.1 (95 % CI 1.1, 26.8; *p* = 0.0351), 6.8 (95 % CI 1.2, 45.4; *p* = 0.0281) and 8.8 (95 % CI 1.5, 63.9; *p* = 0.0151), respectively. However, adherence was not associated with the CMR rate at the 18th month regardless of the cut-off points to define adherence. Adherence was associated with a lower mortality rate when MPR is over 95 % (HR 0.1, 95 % CI 0.01, 0.6; *p* = 0.0118) and 90 % (HR 0.1; 95 % CI 0.01, 0.6; *p* = 0.0118) (Fig. 5).

## Discussion

This study found that most CML patients were highly adherent to imatinib treatment based on the MPR measure, and achieved CCyR at the 18th month; but a minority (4 %) of patients presented a problematic pattern of multiple switches. Regardless of patients’ initial disease phase, adherence to imatinib was associated with a better survival rate and most clinical indicators. The interruptions and patients’ treatment pathway examined in this study reveal the complex and multifaceted nature of CML treatment.

Medicine adherence has been reported [18] to be associated with patients [19], social and medical support, and medication related factors [20]. The use of imatinib for treating CML is likely to be interrupted for various clinical reasons (e.g. efficacy, safety, and tolerability) or accessibility and affordability problems. As imatinib is covered by the NHI in Taiwan, affordability is a less important concern. The higher proportion of patients in the non-adherence group who received interferon prior to imatinib and experienced imatinib-related side effects (Table 2) indicated that patients’ pre-treatment condition and intolerance to imatinib-related side effects are the main reasons of non-adherence to imatinib.

Previous literature has suggested that about 6 % of CML patients were intolerant to the side effects of imatinib [21]. Dose adjustment, temporal interruption, and switching to second-generation TKIs or HSCT are recommended when imatinib intolerance or resistance occurs [7]. However, these treatment alterations may adversely affect therapeutic outcomes [8], and the long-term effectiveness of changing therapeutic schedules is still inconclusive [12].

This study indicates that 26.9 % of patients showed poor adherence to imatinib, this finding is consistent with previous research suggesting that the proportion of poor adherence to imatinib is between 26.4 and 36.1 % [11, 13]. In contrast to the mean MPR ranging from 77.7 to 95.3 % in previous studies [10, 11, 13], the median MPR of this study was 98.3 % (mean 89.7 %). This can be explained by the different study population, sample size and most importantly, the adherence measures.

Currently, there is no generally accepted gold standard for measuring adherence [22] since there is no direct method of measuring imatinib or its metabolites’ levels [12]. So far self-reported measures (e.g. visual analogue scale) [13], Basel Assessment of Adherence Scale [13] and pill count [23] have been found to either over- [24] or under-estimated poor adherence [11, 23, 25, 26]. Patients’ MPR derived from dispensing data for reimbursement purpose has been used to measure adherence under the assumption that patients take medication as dispensed. However, previous studies for assessing imatinib adherence using medical claims data [10, 14] have been limited to chronic phase CML patients using imatinib as the first-line therapy for less than 2 years [11], yet only short-term molecular responses instead of long-term disease progression [11, 12] or survival rates [11, 13] were evaluated.

Although our study has found that patients achieving the therapeutic targets at the beginning of imatinib therapy (the 3rd or 6th month) are likely to achieve the completed therapeutic responses at the 18th month, however a minority of patients could not achieve the therapeutic target (3.4 %). In addition, the proportion of patients achieving CCyR at the 12th month in our study was lower (65 %) compared to previous randomised controlled trials in which either chronic phase patients (69 %) [27] or a higher dose (70 % of started with 800 mg daily) of imatinib (75 %) [28] were involved.

Although a conventional cut-off of 90 % MPR was used in most literature as a proxy to measure adherence of imatinib users [11], various definitions were used to assess MPR, and the clinical implication of adherence defined by this measure is still controversial. Using the sensitivity analysis, we found that MPRs at more than 80, 85 and 90 % was associated with achieving CCyR and MMR at the 18th month and a lower mortality risk. However, when the cut-off point of MPR reached 95 %, then adherence was not associated with any benefit on the proportion of patients achieving clinical outcome indicators. This indicates that an MPR at 95 % may be the ceiling for optimal adherence.

This study longitudinally retrieved details of patients with various disease conditions and previous treatments from medical charts for further analysis, and found direct association between adherence to imatinib and long-term survival rate. However, as this study only included patients from one centre, the results cannot be generalised to a wider population due to its limited sample size. It also assumed that the dispensing records represented actual consumption and therefore, MPR may have over-estimated imatinib adherence. In addition, for those who had switched to second generation TKIs, the imatinib MPR might be relatively lower. During the study period, 26 patients were found to have missed the follow-up appointments. Consequently, they were not included in the analysis of clinical indicators measured at the 18th month of imatinib treatment, and thus the assessment of adherence-related clinical outcomes might be biased. Relevant covariates were included in the regression analysis, although other confounding factors or indication bias may have influenced the results. The comparatively small number of outcomes (deaths) also resulted in relatively wide confidence intervals for the estimate of the strength of association.

## Conclusions

This retrospective, single-centre study has revealed that most of the Asian CML patients were generally well adherent to imatinib treatment despite a minority of them having experienced repeat interruptions. Imatinib adherence is associated with improvement in certain short-term clinical indicators and survival. Further research is needed to validate the adherence measures, to explore the patient and healthcare provider related factors on adherence and evaluate the clinical outcomes for patients switching from imatinib to second generation TKIs in clinical practice.
